# Adjuvant medial versus entire supraclavicular lymph node irradiation in high-risk early breast cancer (SUCLANODE): a protocol for a multicenter, randomized, open-label, phase 3 trial

**DOI:** 10.1186/s12885-024-11831-8

**Published:** 2024-01-09

**Authors:** Li Zhang, Xin Mei, Zhigang Hu, Bo Yu, Chaoyang Zhang, Yong Li, Kaitai Liu, Xuejun Ma, Jinli Ma, Xingxing Chen, Jin Meng, Wei Shi, Xiaofang Wang, Miao Mo, Zhimin Shao, Zhen Zhang, Xiaoli Yu, Xiaomao Guo, Zhaozhi Yang

**Affiliations:** 1 Department of Radiation Oncology, Fudan University Shanghai Cancer Center, 270 Dong-An Road, Shanghai, 200032 China; 2 Department of Oncology, Shanghai Medical College, Fudan University, Shanghai, 200032 China; 3 Shanghai Key Laboratory of Radiation Oncology, Shanghai, 200032 China; 4 Department of Radiation Oncology, The First Affiliated Hospital of USTC, Division of Life Sciences and Medicine, University of Science and Technology of China, Hefei, Anhui 230001 China; 5 Department of Radiotherapy, the Affiliated Jiangyin Hospital of Nantong University, Jiangyin, 214400 China; 6 Department of Radiation Oncology, The First Hospital of Quanzhou Affiliated to Fujian Medical University, Fuzhou, China; 7 Department of Oncology, Guizhou Provincial People’s Hospital, Guiyang, China; 8 Department of Radiation Oncology, The Affiliated Lihuili Hospital of Ningbo University, Ningbo, Zhejiang 315040 China; 9 Department of Cancer Prevention & Clinical Statistics Center, Fudan University Shanghai Cancer Center, Shanghai, 200032 China; 10 Department of Breast Surgery, Fudan University Shanghai Cancer Center, Shanghai, 200032 China; 11 Key Laboratory of Breast Cancer in Shanghai, Fudan University Shanghai Cancer Center, Shanghai, 200032 China; 12 Department of Medical Oncology, Kashgar Prefecture Second People ’ s Hospital, Xinjiang Uyghur Autonomous Region, Kashgar, 844000 China

**Keywords:** Breast cancer, Supraclavicular nodal irradiation, Target volume, Survival outcome, Toxicity

## Abstract

**Background:**

Supraclavicular nodal (SCL) irradiation is commonly used for patients with high-risk breast cancer after breast surgery. The Radiation Therapy Oncology Group (RTOG) and European Society for Radiotherapy and Oncology (ESTRO) breast contouring atlases delineate the medial part of the SCL region, while excluding the posterolateral part. However, recent studies have found that a substantial proportion of SCL failures are located in the posterolateral SCL region, outside of the RTOG/ESTRO-defined SCL target volumes. Consequently, many radiation oncologists advocate for enlarging the SCL irradiation target volume to include both the medial and posterolateral SCL regions. Nevertheless, it remains uncertain whether adding the posterolateral SCL irradiation improves survival outcomes for high-risk breast cancer patients.

**Methods:**

The SUCLANODE trial is an open-label, multicenter, randomized, phase 3 trial comparing the efficacy and adverse events of medial SCL irradiation (M-SCLI group) and medial plus posterolateral SCL irradiation (entire SCL irradiation, E-SCLI group) in high-risk breast cancer patients who underwent breast conserving-surgery or mastectomy. Patients with pathological N2-3b disease following initial surgery, or clinical stage III or pathological N1-3b if receiving neoadjuvant systemic therapy, are eligible and randomly assigned (1:1) to M-SCLI group and E-SCLI group. Stratification is by chemotherapy sequence (neoadjuvant vs. adjuvant), T stage (T3-4 vs. T1-2), N stage (N1-2 vs. N3), and ER status (positive vs. negative). Other radiation volumes are identical in the two arms, including breast/chest wall, undissected axillary lymph node, and internal mammary node. Advanced intensity modulated radiation therapy (IMRT), volumetric modulated arc therapy (VMAT), or tomotherapy techniques are recommended. Both hypofractionated and conventional fractionation schedules are permitted. The primary end point is invasive disease-free survival, and secondary end points included overall survival, SCL recurrence, local-regional recurrence, distance recurrence, safety outcome, and patient-reported outcomes. The target sample size is 1650 participants.

**Discussion:**

The results of the SUCLANODE trial will provide high-level evidence regarding whether adding posterolateral SCL irradiation to medial SCL target volume provides survival benefit in patients with high-risk breast cancer.

**Trial registration:**

ClinicalTrials.gov Identifier: NCT05059379. Registered 28 September 2021, https://www.clinicaltrials.gov/ct2/show/NCT05059379.

## Background

Numerous randomized clinical trials and meta-analyses have demonstrated that radiotherapy significantly reduces local-regional recurrence and improves disease-free survival and breast cancer-specific survival in high-risk breast cancer patients after primary breast surgery [1–4]. Additionally, patients with pathologically positive lymph node after neoadjuvant chemotherapy and surgery have been shown to have high risk of recurrence and could benefit from postoperative radiotherapy [5]. Based on these evidences, it is recommended that patients with pathologically stage III disease after primary breast surgery, as well as those with pathologically positive lymph nodes following neoadjuvant systemic therapy and breast surgery, should receive postoperative breast/chest wall and regional nodal irradiation (RNI).

The supraclavicular nodal (SCL) region is the most frequent site of regional nodal failure after mastectomy and breast-conserving surgery, thus remained an essential component of RNI [6]. In the era of traditional 2-dimensional (2D) radiotherapy, the SCL field was defined based on anatomical boundaries. Typically, the lateral border was located lateral to humeral head with a block to shield the humeral head or medial 2/3 clavicle. Typical radiation dose coverage includes only medial part of SCL region, not extending to the posterolateral SCL fossa [3, 4]. In the modern radiotherapy era, with the advent of computed-tomography (CT)-based radiation treatment planning, atlas consensus was proposed by the Radiation Therapy Oncology Group (RTOG) in 2009 and the European Society for Radiotherapy and Oncology (ESTRO) in 2015 [7, 8]. The RTOG define the dosal and lateral boundaries of the SCL clinical target volume (CTV) at the anterior aspect of the scalene muscle and lateral edge of sternocleidomastoid muscle (cranial) and the junction of the 1st rib and clavicle (caudal), respectively [7]. The ESTRO define the dosal and lateral boundaries of the SCL CTV at pleura and including the anterior scalene muscles and connecting to the medial border of axillary level 3, respectively [8]. Therefore, both RTOG and ESTRO consensus still encompass only the medial part of SCL region.

However, several recent studies have focused on mapping the locations of the SCL metastases and have demonstrated a notable SCL metastases occurred in the overlooked posterolateral part of SCL region (14 ~ 82%), thus outside of the RTOG and ESTRO-defined SCL CTVs [9–13]. Consequently, a growing number of radiation oncologists advocate for enlarging SCL CTV to include both medial and posterolateral SCL region, creating what is referred to as the “entire SCL region”. In the context of ongoing clinical trials, the RADCOMP atlas for RTOG 3509/3510 trial has introduced the posterolateral SCL as an optional volume to consider for irradiation [14]. Additionally, a phase 3 randomized clinical trial exploring the impact of internal mammary nodal irradiation on disease-free survival in high-risk breast cancer patients in currently recruited in China, with the SCL CTV encompassing the entire SCL region [15]. However, some other clinical trials, such as NRG 9353, Alliance A11022, Danish Breast Cancer Cooperative Group (DBCG) RT Recon Trial, and DBCG HYPO II Trial still define the SCL CTV based on RTOG atlas or ESTRO guidelines. Therefore, controversy exists in practice regarding the optimal definition of the SCL target volume. Recently, TransAtlantic Radiation Oncology Network conducted a review and recommended that SCL CTV should follow the RTOG or the RADCOMP atlas for patients at high and very high regional recurrence risk [16].

We previously conducted a retrospective study comparing cancer outcomes and toxicities between medial SCL irradiation versus entire SCL irradiation in 544 patients with clinical or pathological stage N2-3b breast cancer at our institution from January 2015 to December 2016. With a median follow-up time of 64.2 months, we found no significant differences in the cumulative incidence of SCL recurrence, disease-free survival, or overall survival between the two groups [17]. However, high-level evidence is lacking. The current SUCLANODE trial is a randomized trial investigating whether adding posterolateral SCL region to the medial SCL target volume will improve disease-free survival in patients with high-risk early breast cancer. Additionally, acute and late radiation-related toxicities and quality of life will be evaluated, with a particular focus on shoulder function and pain. The study protocol for the SUCLANODE trial is described in this article.

## Methods/design

### Study design

**Fig. 1:**
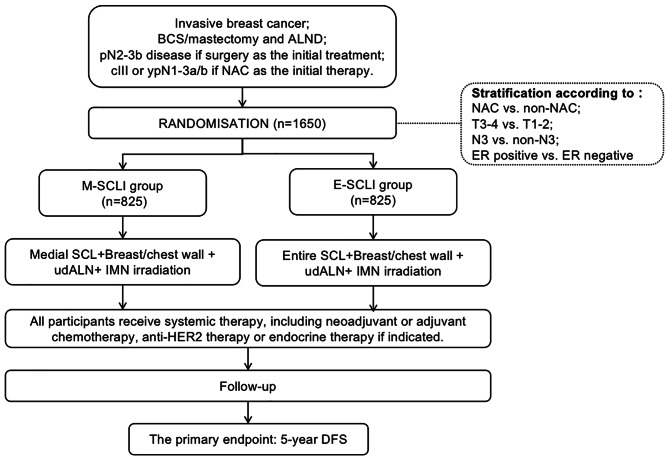
Flow chart of the SUCLANODE trial. Abbreviations: BCS = breast conserving-surgery; ALND = axillary lymph node dissection; M-SCLI = medial supraclavicular lymph nodal irradiation; E-SCLI = entire supraclavicular lymph nodal irradiation; NAC = neoadjuvant chemotherapy; udALN = undissected axillary lymph node dissection; IMN = internal mammary node; SCL = supraclavicular lymph node

The SUCLANODE trial is an open label, multicenter, randomized, phase 3 trial comparing the efficacy and adverse events of medial SCL irradiation (M-SCLI group) versus entire SCL irradiation (E-SCLI group) in patients with high-risk early breast cancer after breast conserving-surgery or mastectomy. High-risk is defined as follows: pN2-3b disease if surgery was the initial treatment, or cIII or ypN1-3a/b if neoadjuvant systemic therapy was the initial therapy. The target sample size is 1650 participants. We hypothesize that E-SCLI, which includes the posterolateral SCL fossa in the SCL CTV, provide superior disease-free survival compared to M-SCLI group. The Flow chart of this trial is summarized in Fig. 1.

### Primary endpoint

The primary endpoint is disease-free survival (DFS), defined as the time from the date of randomization to the date of the first occurrence of any following events: ipsilateral invasive local recurrence (including ipsilateral breast tumor recurrence, ipsilateral chest wall recurrence), ipsilateral invasive regional recurrence (including axillary, supraclavicular, or internal mammary nodes), distant recurrence, contralateral invasive breast cancer, or death from any cause.

### Secondary endpoints

1. Overall survival: Defined as the time from randomization to death from any cause.
2. SCL lymph node recurrence: Defined as the time from randomization to the date of the first ipsilateral supraclavicular lymph node recurrence.
3. Local-regional recurrence: Defined as the time from randomization to the date of the first ipsilateral breast, chest wall, axillary, supraclavicular, or internal mammary nodal recurrence.
4. Distance recurrence: Defined as the time from randomization to the date of the first distant breast cancer recurrence.
5. Safety outcomes: The frequency and severity of acute and late radiation-related adverse events are assessed and graded based on the Common Terminology Criteria for Adverse Events (CTCAE) Version 4.0, or RTOG/the European Organization for Research and Treatment of Cancer (EORTC) late radiation morbidity scale and LENT-SOMA criteria if CTCAE grading not available.
6. Patient-reported outcomes: Assessment of treatment-related symptoms, health-related quality of life via the EORTC Quality of Life Questionnaire (EORTC-QLQ-C30; version 3) and breast cancer module (QLQ-BR23), and upper limb function via the Quick Disabilities of the Arm, Shoulder and Hand (q-DASH) questionnaire.

### Exploratory outcome

To explore whether radiotherapy resistance mechanisms can be identified through analysis of tumor and blood sample derivatives. Future exploratory research into factors that may influence efficacy and safety may be performed on the collected and stored tumor and blood samples. Efficacy outcomes considered for this analysis will include local-regional recurrence and DFS, as appropriate.

### Inclusion criteria

1. Female subjects aged 18 ~ 75 yeas on the day of signing informed consent.
2. Histologically confirmed invasive breast cancer.
3. Have undergone breast conserving-surgery or total mastectomy (including breast reconstruction) with negative margins and axillary lymph node dissection (≥ 10 lymph nodes dissected). Axillary level 3 dissection and internal mammary node dissection are not required but may be performed per surgeon’s discretion.
4. Clinical or pathological stage: pathological N2-3b disease if surgery was the initial treatment; initial clinical stage III or pathological N1-3b if neoadjuvant systemic therapy was the initial therapy.
5. Completion of neoadjuvant or adjuvant systemic treatment (taxane and/or anthracycline-based) consisting of at least 6 cycles.
6. Will receive endocrine therapy for at least 5 years for estrogen receptor (ER) and/or progesterone receptor (PR) positive patients; will receive anti-HER2 (human epidermal growth factor receptor 2) therapy for 1 year for HER2 positive patients.
7. Radiation therapy must begin no later than 12 weeks after the last breast cancer surgery or the last dose of adjuvant chemotherapy.
8. Eastern Cooperative Oncology Group (ECOG) performance status of 0 or 1.
9. Acquirement of informed consent.

### Exclusion criteria

1. Clinical stage N3c at presentation (metastatic supraclavicular lymph node).
2. Clinical stage T4 at presentation and failed to downstage after neoadjuvant systemic therapy.
3. Stage IV (metastatic) breast cancer.
4. ECOG performance status ≥ 2.
5. History of any prior ipsilateral or contralateral breast cancer.
6. Positive sentinel lymph node without further axillary lymph node dissection.
7. History of prior radiation therapy.
8. Current pregnancy and/or lactation.
9. History of other malignancies except for appropriately treated skin basal cell carcinoma and cervical carcinoma in situ.
10. Inability to tolerate chemotherapy, anti-HER2 therapy, or endocrine therapy.
11. Current severe, uncontrolled systemic disease (e.g., clinically significant cardiovascular, pulmonary, hepatic, renal, hematologic, or psychiatric disease).
12. Inability or unwillingness to comply with protocol requirements.

### Randomization

Eligible patients will be centrally randomized in a 1:1 ratio to the M-SCLI group and the E-SCLI group. Randomization will be stratified according to the following factors: neoadjuvant systemic therapy vs. adjuvant systemic therapy; T3-4 vs. T1-2; N3 vs. non-N3; ER positive vs. ER negative.

### Treatment arms

Trial participants will be allocated to two treatment groups:

1. M-SCLI group (standard arm): The radiation target volume includes ipsilateral medial SCL region, breast/chest wall, undissected axillary lymph nodes, and internal mammary nodes.
2. E-SCLI group (experimental arm): The radiation target volume includes ipsilateral medial plus posterolateral SCL regions (also called entire SCL region), breast/chest wall, undissected axillary lymph nodes, and internal mammary nodes.

### Radiation therapy

#### Localization, simulation, and immobilization

Patients should be optimally positioned supine using breast boards, wing boards and/or other immobilization devices per treating physician’ discretion. Radio-opaque markers will be placed on the skin at scar, inframammary fold, and superior border of the breast tissue. A treatment planning CT scan will be performed with an image thickness of ≤ 0.5 cm.

#### Target and normal tissue volume definitions

##### Clinical target volumes (CTVs)

The CTVs include ipsilateral chest wall/breast, supraclavicular lymph nodes, undissected axilla (including interpectoral lymph nodes), and internal mammary nodes, with a tumor bed boost to the lumpectomy cavity for patients who underwent breast-conserving surgery. For patients who received mastectomy, a boost to the scar is delivered only when indicated. If positive internal mammary nodes are identified on initial CT and/or MRI, a nodal boost may be delivered at the discretion of the treating physician. In general, CTV delineation will follow the RTOG and ESTRO guidelines [7, 8, 18]. However, for some controversial areas, modifications have been made at Fudan University Shanghai Cancer Center to establish institutional guidelines for CTV delineation. The suggested CTV delineation for breast, chest wall, and lymph node regions is provided in Table 1.

**Table 1:** Suggested CTV delineation for breast, chest wall, and lymph node regions

			Cranial	Caudal	Ventral	Dorsal	Medial	Lateral
Breast CTV (CTV_B)			Upper border of palpable/visible breast tissue; maximally up to the caudal edge of the sterno-clavicular joint	Most caudal CT slice with visible breast	5 mm under skin surface (3 mm for small breast)	Major pectoral muscle or costae and intercostal muscles where no muscle	Lateral to the medial perforating mammarian vessels; maximally to the edge of the sternal bone	Lateral breast fold; anterior to the lateral thoracic artery
Chest wall CTV (CTV_CW)			Guided by palpable/visible signs; if appropriate guided by the contralateral breast; maximally up to the caudal edge of the sterno-clavicular joint	Guided by palpable/visible signs; if appropriate guided by the contralateral breast	Skin	Major pectoral muscle or costae and intercostal muscles where no muscle (For T4a/T4c cases, the major pectoral muscle and ribs should be included)	Guided by palpable/visible signs; if appropriate guided by the contralateral breast	Guided by palpable/visible signs; if appropriate guided by the contralateral breast. Usually anterior to the mid-axillary line
Supraclavicular CTV	Medial supraclavicular CTV (CTV_M_SCL)		Caudal to the cricoid cartilage	Includes the subclavian vein with 5 mm margin, thus connecting to the cranial border of CTVn_IMN	Sternocleidomastoid muscle, dorsal edge of the clavicle	Anterior aspect of the scalene muscle	Including the jugular vein without margin; excluding the thyroid gland and the common carotid artery	Cranial: Sternocleidomastoid muscle Caudal: junction of 1st rib and clavicle
	Entire supraclavicular CTV (CTV_E_SCL)		Same as above	Same as above	Same as above	Trapezius muscle	The same as CTV_M_SCL anteriorly, avoids the scalene and elevator scapulae muscles posteriorly	Sternocleidomastoid muscle, clavicle, and connecting to axillary level III
Internal mammary node CTV (CTV_IMN)			Caudal limit of CTV_SCL	Cranial side of the 4th rib (in selected cases 5-6th rib)	Ventral limit of the vascular area	Pleura	5 mm from the internal mammary vein (artery in cranial part down to and including first intercostal space)	5 mm from the internal mammary artery
Undissected axillary CTV (CTV_udALN)		Undissected Axillary level 2	Includes the cranial extent of the axillary artery (i.e. 5 mm cranial of axillary vein)	The caudal border of the minor pectoral muscle, but should exclude the extent of the dissection	Minor pectoral muscle	Up to 5 mm dorsal of axillary vein or to costae and intercostal muscles	Medial edge of minor pectoral muscle	Lateral edge of minor pectoral muscle, but should exclude the extent of the dissection
		Axillary level 3	Includes the cranial extent of the subclavian artery (i.e. 5 mm cranial of subclavian vein)	5 mm caudal to the subclavian vein	Major pectoral muscle	Same as level 2	Junction of subclavian and internal jugular veins, connects to CTV_SCN	Medial side of the minor pectoral muscle
		Interpectoral nodes	Includes the cranial extent of the axillary artery (i.e. 5 mm cranial of axillary vein)	The caudal border of the minor pectoral muscle	Major pectoral muscle	Minor pectoral muscle	Medial edge of minor pectoral muscle	Lateral edge of minor pectoral muscle

Abbreviations: CTV=clinical target volume; CT=computer tomography

**Fig. 2:**
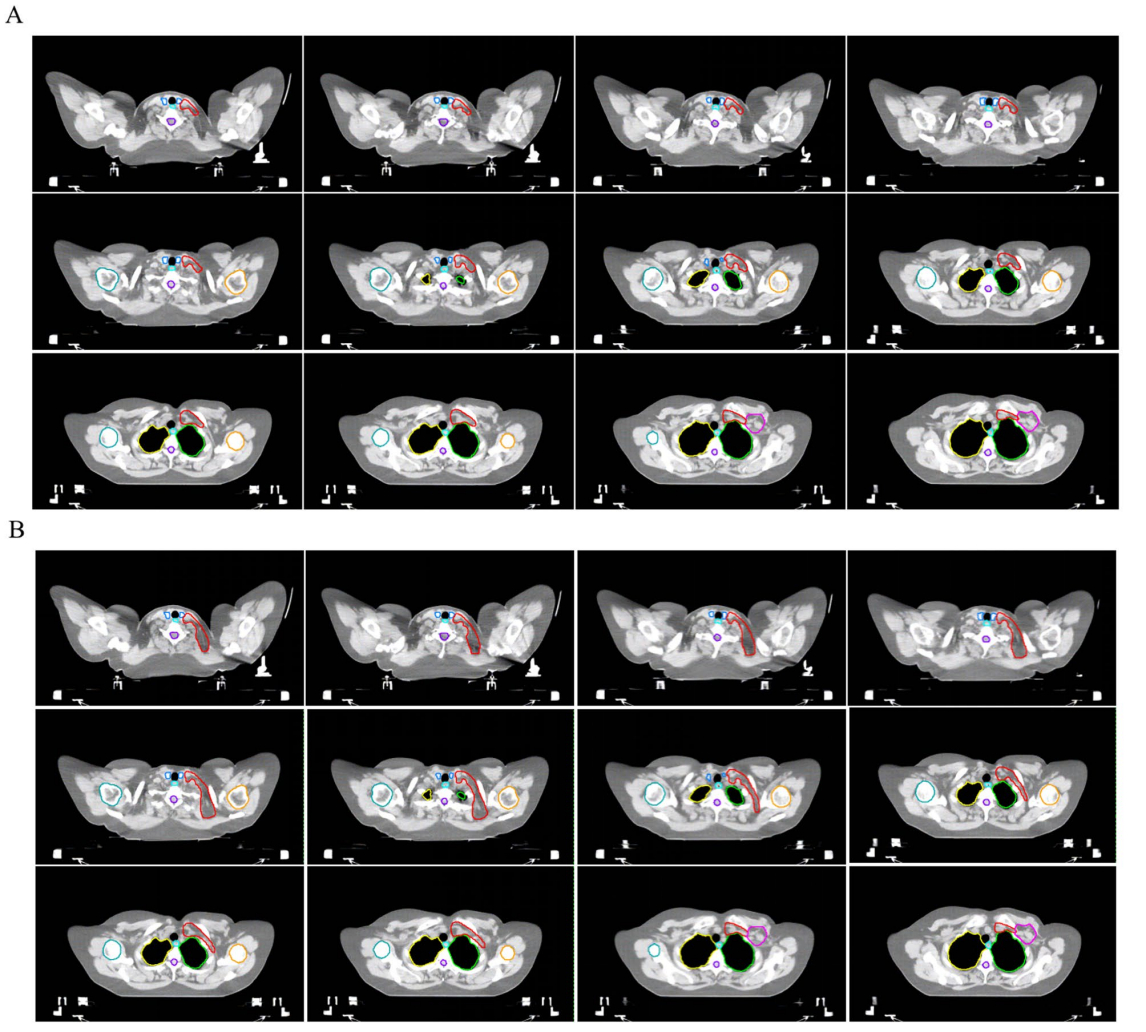
Atlas of medial supraclavicular CTV (CTV_M_SCL) and entire supraclavicular CTV (CTV_E_SCL). **A**: CTV_M_SCL (red). **B**: CTV_E_SCL (red). Abbreviations: CTV = clinical target volume

1. *Tumor bed (TB)*: Contoured using all available clinical and radiographic information, including surgical clips, excision cavity volume, seroma, architectural distortion, lumpectomy scar.
2. *Tumor bed CTV (CTV_TB)*: Defined as 1 cm 3D expansion around the TB. Limit dorsal border at anterior surface of the pectoralis and/or serratus anterior muscles, and ventral border at 5 mm under skin surface.
3. *Breast CTV (CTV_B)*: Same as the definitions of the ESTRO breast cancer consensus guidelines.
4. *Chestwall CTV (CTV_CW)*: Same as the definitions of the ESTRO breast cancer consensus guidelines, except for the ventral border is skin due to thin postoperative chest wall in Chinese patients. For T4a/T4c cases, the major pectoral muscle and ribs should be included. The surgical scar is routinely included in the CTV_CW.
5. *Supraclavicular CTV*.* Medial supraclavicular CTV (CTV_M_SCL)*: Follows the definitions of RTOG or ESTRO breast cancer consensus guidelines. The cranial border is caudal to the cricoid cartilage; the caudal border is the junction of the internal jugular vein and subclavian vein; the ventral border is sternocleidomastoid muscle and dorsal edge of the clavicle; the dorsal border is anterior aspect of the scalene muscle (anterior and medial); the medial border includes the internal jugular vein and excludes the thyroid gland and the common carotid artery; the lateral border is sternocleidomastoid muscle (cranial), junction of the first rib and clavicle (caudal). The atlas of CTV_M_SCL is shown in Fig. 2A.*Entire supraclavicular CTV (CTV_E_SCL)*: The cranial, caudal, and ventral borders are the same as the definitions of CTV_M_SCL. The dorsal border is trapezius muscle; the medial border is the same as CTV_M_SCL anteriorly, avoids the scalene and elevator scapulae muscles posteriorly; the lateral border is sternocleidomastoid muscle, clavicle, and connecting to axillary level III. The atlas of CTV_E_SCL is shown in Fig. 2B.
6. *Internal mammary node CTV (CTV_IMN)*: Same as the definitions of the ESTRO breast cancer consensus guidelines, including the internal mammary vessels in the first three intercostal spaces. The caudal limit of CTV_IMN is typically the cranial side of the 4th rib, but might be individually extended by one or two more intercostal space per treating physician’ discretion for patients with metastatic internal mammary nodes or based on tumor location and burden.
7. *Undissected axillary CTV (CTV_udALN)*: Since axillary dissection typically removes level 1–2 axillary nodes. CTV_udALN consists of the undissected portions of axilla, typically including level 3, some of axillary level 2, and interpectoral nodes. The caudal and lateral borders are the most cranial and medial extent of the dissection, other anatomical borders follow the definitions of the ESTRO breast cancer consensus guidelines for axillary levels.

##### Planning target volumes (PTVs)

PTVs are generated by adding 5 mm uniform margins to the CTVs, including PTV_TB, PTV_B, PTV_CW, PTV_SCL, PTV_IMN, and PTV_unALN. For patients who underwent breast-conserving surgery, additional PTV_B_EVA and PTV_TB_EVA are generated by limiting the ventral border of PTV_B and PTV_TB at 5 mm beneath the skin surface for dose volume histogram (DVH) evaluation. Similarly, for patients who underwent mastectomy, an additional PTV_CW_EVA is generated by limiting the ventral border of PTV_CW at skin surface for DVH evaluation.

##### Organs at risk (OARs)

Normal structures include heart, left anterior descending, ipsilateral lung, contralateral lung, contralateral breast, humeral head, thyroid, esophagus, and brachial plexus. Of these structures, left anterior descending are optional.

#### Dose specifications

Both hypofractionated and conventional fractionated radiation therapy are permitted: the conventional fractionated regimen is 50 Gy in 25 fractions of 2 Gy; the hypofractionated schedule is 42.5 Gy in 16 fractions of 2.67 Gy. Tumor bed or scar boost is at the discretion of the treating physician, boost doses will be 10 ~ 16 Gy in 5 ~ 8 fractions of 2 Gy, for total cumulative doses of 60 ~ 66 Gy.

#### Technique factors and treatment planning

3D-conformal radiation therapy (3D-CRT) techniques are not recommended due to significant incidental irradiation of the SCL region. Advanced intensity modulated radiation therapy (IMRT), volumetric modulated arc therapy (VMAT), or tomotherapy techniques are recommended instead. Methods to minimize the cardiac exposure such as gating or breath-holding are permitted per treating physician’ discretion.

Suggested PTV compliance criteria are as follows: at least 95% of each PTV receives ≥ 95% of the prescribed dose (it is acceptable if ≥ 95% of each PTV receives ≥ 90% of the prescribed dose, except for PTV_IMN, it is acceptable if ≥ 90% of the PTV_IMN receives ≥ 90% of the prescribed dose); the maximum point dose (volume that is 0.03 cm^3^) is recommended to be ≤ 115% of the prescription dose (≤ 120% acceptable), and should be evaluated without the contribution of the boost fields; ≤ 35% of the PTV_B_EVA, PTV_CW_EVA, or PTV_IMN receives 100% of the boost prescribed dose when a boost is included in the composite plan (≤ 40% acceptable); ≤ 5% of the PTV_TB_EVA receives ≥ 110% of the boost prescribed dose (≤ 10% acceptable).

The OARs dose constraints are shown in Table 2.

**Table 2:** Suggested dose constraints of organs at risk

	Conventional fractionated RT (50 Gy in 25 fractions)		Hypofractionated RT (42.56 Gy in 16 fractions)	
	required	acceptable	required	acceptable
Heart(Left_sided)	Dmean ≦ 6 Gy	Dmean ≦ 8 Gy	Dmean ≦ 5.1 Gy	Dmean ≦ 6.8 Gy
	V20Gy ≦ 5%	V25Gy ≦ 5%	V17Gy ≦ 5%	V21Gy ≦ 5%
Heart(Right_sided)	Dmean ≦ 2 Gy	Dmean ≦ 3 Gy	Dmean ≦ 1.7 Gy	Dmean ≦ 2.6 Gy
	V20Gy ≦ 0%	V25Gy ≦ 0%	V17Gy ≦ 0%	V21Gy ≦ 0%
Ipsilateral Lung	V20Gy ≦ 30%	V20Gy ≦ 35%	V17Gy ≦ 30%	V17Gy ≦ 35%
	V5Gy ≦ 60%	V5Gy ≦ 70%	V4.2 Gy ≦ 60%	V4.2 Gy ≦ 70%
Contralateral lung	V5Gy ≦ 10%	V5Gy ≦ 15%	V4.2 Gy ≦ 10%	V4.2 Gy ≦ 15%
Contralateral breast	V5Gy ≦ 10%	V5Gy ≦ 15%	V4.2 Gy ≦ 10%	V4.2 Gy ≦ 15%
Thyroid	Dmean ≦ 25 Gy	Dmean ≦ 26.2 Gy	Dmean ≦ 21.2 Gy	Dmean ≦ 22.2 Gy
Esophagus	V20Gy ≦ 10%	V20Gy ≦ 15%	V17Gy ≦ 10%	V17Gy ≦ 15%
Spinal cord	Dmax ≦ 45 Gy		Dmax ≦ 38.2 Gy	
Humeral head	Dmean ≦ 25 Gy	Dmean ≦ 26.2 Gy	Dmean ≦ 21.2 Gy	Dmean ≦ 22.2 Gy
	V30Gy ≦ 30%	V30Gy ≦ 35%	V28.5 Gy ≦ 30%	V28.5 Gy ≦ 35%
Brachial plexus	Dmax ≦ 54 Gy	Dmax ≦ 56.7 Gy	Dmax ≦ 46 Gy	Dmax ≦ 48.3 Gy

V_XXGy_ ≦YY%: the volume of each organs at risk receiving XXGy should be no more than YY%

#### Treatment verification

Before the first time of radiotherapy, portal films or 3D-CRT images (e.g., conebeam CT, MV CT, kV CT) must be obtained and approved by a physician. During radiotherapy, portal films or 3D-CRT images must be obtained every 5 fractions.

### Follow-up

During radiotherapy, patients will attend the clinic every 2 weeks and at the end of treatment. Physical examination, hematology tests, and acute radiation toxicity (including acute radiation dermatitis, pneumonitis, esophagitis, neck and should pain, and myocardial ischemia/infarction) will be performed.

After completing radiotherapy, efficacy assessments for recurrence, new cancers and overall survival will be conducted every 3 months for the first 2 years, every 6 months for the years 3 ~ 5, and then annually until 10 years from randomization. Efficacy follow-up includes symptoms assessment, physical examination, hematology tests, blood chemistry/tumor-markers tests, Ultrasounds, mammogram annually, and chest CT scans annually. Breast magnetic resonance imaging (MRI) is permitted in doubtful cases. Additional imaging such as positron emission tomography /computed tomography (PET/CT), bone emission computed tomography (ECT), brain MRI, or biopsy will be performed as indicated for suspected recurrence or metastasis. Acute radiation adverse events will be assessed until 3 months after completing radiotherapy. Late radiation adverse events will be assessed starting at 3 months after completing radiotherapy and then annually. The follow-up workflow is shown in Table 3.

**Table 3:** Follow-up workflow

	Baseline	During RT	End of RT	First 2 years after RT	3 ~ 5 years after RT	Beyond 5 years after RT
		Every 1 ~ 2 week		Every 3 months	Every 6 months	Every 12 months
Clinical examination	X	X	X	X	X	X
Hematology tests	X	X	X	X	X	X
Blood chemistry/tumor-markers tests	X			X	X	X
Ultrasounds	X			X	X	X
Mammogram	X			every 12 months after RT		
Chest CT*	X			every 12 months after RT		
Acute radiation adverse events	X	X	X	At 2 weeks and 3 month after RT		
Late radiation adverse events	X			At 3 month after RT, and than every 12 months		
PROs/QoL questionnaires	X			At 3 month after RT, and than every 12 months		
Blood biomarker samples	X		Carried out if possible			

* Additional imaging such as positron emission tomography /computed tomography (PET/CT), bone emission computed tomography (ECT), brain (MRI), or biopsy will be performed as indicated for suspected recurrence or metastasis

Abbreviations: RT = radiation therapy; CT = computer tomography; MRI = magnetic resonance imaging; PROs = patient reported outcomes; QoL = quality of life

### Sample size and statistical analysis

The primary endpoint is DFS. According to data from a Chinese prospective study, the 5-year DFS for high-risk patients after breast-conserving surgery or mastectomy is 73% [19] (control group). We assume a clinically meaningful difference in 5-year DFS of 5% between groups. The hypothesis of the current study is that the 5-year DFS will be superior in the E-SCLI group (experiment group) compared to the M-SCLI group (control group), with a 5-year DFS increase from 73% in the M-SCLI group to 78% in the E-SCLI group. Sample size calculation for comparing DFS was performed with a two-sided α = 0.05 and 80% power. Hazard ratio is 0.79. This yields to a total sample size of 1500 patients. Accounting for a 10% dropout rate, the total sample size is increased to 1650 patients (825 per group). Based on these assumptions, it is estimated that 510 events will be required for the final analysis. Statistical analysis will be performed based on the intent-to-treat principle, with all patients analyzed as randomized, regardless of eligibility or protocol compliance.

### Ethics

The trial was approved by the fudan university shanghai cancer center ethics committee (2108241-22). The study is registered in ClinicalTrails.gov (NCT04320979).

### Trial status

Patient accrual was started on September 1, 2021 and is currently ongoing.

## Discussion

In the current report, we present the protocol of the SUCLANODE trial, which aims to investigate whether adding the posterolateral SCL irradiation to medial SCL irradiation improves DFS in high-risk breast cancer patients after breast surgery. SCL irradiation is widely used for patients with high-risk breast cancer treated with mastectomy and breast-conserving surgery. However, the optimal SCL target volume has been controversial in recent years, high-level data directly comparing entire versus medial SCL irradiation in high-risk breast cancer patients treated with surgery is lacking.

For high-risk patients with less than N3c disease receiving modern adjuvant systemic therapy and radiotherapy, the risk of posterolateral SCL recurrence may be quite low. In our prior retrospective study comparing medial versus entire SCL irradiation in 544 patients with clinical or pathologic N2-N3b disease after breast surgery, only 2 patients (0.4%) had posterolateral SCL recurrence, including 1 who received entire SCL irradiation and had in-field recurrence. Entire SCL irradiation did not significantly improve SCL control and DFS compared to medial SCL irradiation [17]. Another study by Bazan et al. reported a similarly low 0.4% rate of posterolateral SCL recurrence (1/268) in high-risk breast cancer patients treated with IMRT (168 patients) or 3D-CRT technique (72 patients) [20]. In that study, all CTVs were delineated according to the RTOG breast contouring atlas consensus, thus only the medial SCL region was irradiated. None of the patients in the IMRT group had posterolateral SCL recurrence, indicating a very low risk of recurrence in that region even unintentionally excluded from IMRT treatment volumes in those high-risk patients. The one patient with posterolateral SCL recurrence was treated with 3D-CRT technique, which delivered a mean dose of 4647 cGy to the posterolateral SCL region. This evidence suggests posterolateral SCL recurrence after adjuvant RNI may not be attributable to inadequate target volume coverage.

The biophysical effects of radiotherapy are not tumor-specific and can also cause toxicity due to exposure of surrounding normal tissues. The toxicity is of particular concern in breast cancer as more patients become long-term survivors due to advances in systemic and local therapies. Expanding the SCL target volume will inevitably increase neck and shoulder radiation dose, potentially heightening acute and late normal tissue toxicity and reducing quality of life. Bazan et al. found 3D-CRT technique delivered significantly higher incidental dose to the shoulder and back versus IMRT technique, with a tend toward reduced q-DASH scores indicating less shoulder morbidity in patients treated with IMRT technique (*p* = 0.078) [20]. Our prior study also observed a significantly higher rate of pain in the E-SCLI group compared to M-SCLI (26.6% vs. 15.1%, *p* = 0.005) [17]. In this trial, we will evaluate shoulder function via q-DASH questionnaire, patients-reported shoulder/back symptoms, and quality of life. Additionally, a previous study showed 3D-CRT technique delivered significantly higher incidental radiation dose to the posterolateral SCL region than IMRT technique (46.7 Gy vs. 30.2 Gy *P* < 0.0001) when treating medial SCL region based on RTOG consensus [20]. We will utilize advanced IMRT, VMAT, or tomotherapy techniques instead of 3D-CRT technique to minimize incidental irradiation to the posterolateral SCL region in the M-SCLI arm.

## Conclusion

In conclusion, the SUCLANODE trial is a superiority, multicenter, randomized controlled trial that compares the efficacy and safety of medial SCL irradiation and entire SCL irradiation in patients with high-risk breast cancer after breast surgery. We anticipate that the results of the current trial will provide high-level evidence to optimize the SCL irradiation target volume.

## Data Availability

Data sharing is not applicable to this article as the current study is still open for inclusion of patients.

## Abbreviations

SCL: Supraclavicular nodal
RTOG: Radiation Therapy Oncology Group
ESTRO: European Society for Radiotherapy and Oncology
M-SCLI: Medial SCL irradiation
E-SCLI: Entire SCL irradiation
IMRT: Intensity modulated radiation therapy
VMAT: Volumetric modulated arc therapy
RNI: Regional nodal irradiation
2D: 2-Dimensional
CT: Computed-tomography
CTV: Clinical target volume
DBCG: Danish Breast Cancer Cooperative Group
DFS: Disease-free survival
CTCAE: Common Terminology Criteria for Adverse Events
EORTC: European Organization for Research and Treatment of Cancer
Q-DASH: Quick Disabilities of the Arm, Shoulder and Hand
ER: Estrogen receptor
PR: Progesterone receptor
HER2: Human epidermal growth factor receptor 2
ECOG: Eastern Cooperative Oncology Group
PTVs: Planning target volumes
DVH: Dose volume histogram
OARs: Organs at Risk
3D-CRT: 3D-conformal radiation therapy
MRI: Magnetic resonance imaging
PET/CT: Positron emission tomography /computed tomography
ECT: Emission computed tomography
